# Affect in Peer Group Learning During Virtual Science Inquiry: Insights From Self-Reports and Video Observations

**DOI:** 10.3389/fpsyg.2019.02981

**Published:** 2020-01-14

**Authors:** Tarja Pietarinen, Simone Volet, Erno Lehtinen, Marja Vauras

**Affiliations:** ^1^Department of Teacher Education and Centre for Learning Research, University of Turku, Turku, Finland; ^2^School of Education, Murdoch University, Murdoch, WA, Australia

**Keywords:** affect, group work, collaboration, science learning, CSCL, virtual learning environment

## Abstract

The purpose of this study was to explore affect in small groups learning together face-to-face in a virtual learning environment. The specific aims of the study were to establish how affect within groups (valence, intensity) related to the quality of group outcome (high, average, low), and to capture individual differences within the groups by using a multimethod approach. Participants were six groups of three high school students (*N* = *18*) who achieved distinct outcome levels. Students’ self-reports of their affect and observed affect (researcher-coded selected segments from videos) were used to examine affect during three phases of interdisciplinary science inquiry, namely, planning the experiment, experimenting in the virtual laboratory, and concluding and preparing a joint group presentation. The overall results showed that positive affect was prevalent in both self-reports and researcher-coded observations across all phases. However, while self-reports displayed a strong dominance of positive affect, there was more variation in observed affect. Furthermore, the intensity of affect was higher in self-reports than in observations, for both positive and negative affect. Nonetheless, no effect of affect on group outcome was found. Finally, while within-group consistency in affect was evident in the extreme groups (high, low performance), it was more ambivalent in the groups that achieved an average performance. The results are discussed in light of the literature, and directions for future research on affect in collaborative learning are proposed.

## Introduction

As evidenced in the literature, science learning is affected by attitudes, interest, and motivation, and the lack of interest toward science domains has been repeatedly remarked in recent decades (Ramsden, 1998; Alsop and Watts, 2003; Ainley and Ainley, 2011). This concern is still present, as interest, motivation, and engagement in science learning are continuously declining (Schneider et al., 2016). To enhance motivation and quality of learning in STEM (science, technology, engineering, and mathematics) subjects, new, engaging learning environments based on technology-supported inquiry and collaboration have been developed (de Jong, 2019). However, findings of the success of collaborative inquiry learning are diverse. Many studies have shown that new learning environments have positive effects on learning and motivation (see review by de Jong, 2019), but other research summaries have questioned the superior effects of inquiry learning (Stockard et al., 2018). In addition, the study of Chang et al. (2017) showed that working in collaborative groups in an inquiry-based environment is not engaging for all students (Chang et al., 2017).

The above described diverse findings of the effects of inquiry learning heighten the importance of studying the role of affect when developing novel learning environments in STEM domains (see also Gegenfurtner et al., 2019). The importance of affect in supporting students’ interest, engagement, achievement, and experiences of science is widely acknowledged (Lin et al., 2012; Sinatra et al., 2014; King et al., 2015). In particular, positive affect has an especially significant impact on science activities (Laukenmann et al., 2003) and achievement (Ahmed et al., 2013; Liu et al., 2014). Students are more likely to feel positive (happy, confident, and successful), and perceive science as important to them if they are appropriately challenged according to their skills (Schneider et al., 2016).

During the last decades, many studies have dealt with affects and emotions in collaborative and technology-supported learning environments (e.g., Järvelä and Hadwin, 2013; Miller and Hadwin, 2015). Computer-supported collaborative (CSCL) inquiry environments have many features that differ from traditional teacher-centered classroom situations (e.g., Roschelle et al., 2011), and studies have shown that there are unique sources and directions of emotions in these environments (Järvenoja and Järvelä, 2005; Wosnitza and Volet, 2005). The unique features of these learning environments mean that the findings of studies focused on affect in traditional classroom contexts mainly emphasizing students’ interpretations of teacher expectations and feedback (e.g., Salonen et al., 1998) are not necessarily directly applicable in the CSCL inquiry environments, which are still rarely used in science teaching in schools. This study focuses on the group, and individual level affects when students study demanding science tasks in a computer simulation-based collaborative inquiry environment. The main aim is to study what kind of affects appear in differently achieving student groups across different phases of inquiry processes by using a multimethod approach.

Collaborative learning environments based on CSCL inquiry consist of many features, which are specific for the appraisal and arousal of affects (Prince, 2004; de Jong, 2019). For example, these environments are assumed to provide students with engaging opportunities for learning by performing meaningful activities, such as modifying or elaborating the content they are studying. Working in these environments also requires reflection of students’ ideas and discussing them with others. In addition, in CSCL inquiry, there is more freedom for the students’ ideas and approaches than in direct teaching. These affordances and requirements of collaborative and technology-supported learning environments can arouse positive affects in some students but could also arouse anxiety and negative affect in others (Chang et al., 2017).

According to Wosnitza and Volet (2005), in technology-supported social learning activities, emotions are typically directed to self, other(s), the task, and the technology; thus, the understanding of affect in technology-enhanced collaborative settings is ambiguous. Thus, some of the emotions are task-related and can be called epistemic emotions (Pekrun et al., 2017), whereas others are focused on social and other aspects of the environments. Emotions are a crucial part of collaborative learning, but it is complicated how they enhance productive engagement (Polo et al., 2016). Collaborative conditions can arouse positive affects, but can also result in conflicts and tension, leading to negative affect (Baker et al., 2013). Social interaction, as an element of a learning environment can create strong emotional responses (Do and Schallert, 2004), which shows that social interaction, and the social context in general, may trigger students’ positive or negative affective reactions and have a strong effect on their engagement (Zschocke et al., 2016).

Many of the studies on collaborative learning in science education have highlighted the positive effects of collaboration and small group work in science learning (e.g., Springer et al., 1999; Shibley and Zimmaro, 2002). Previous research has emphasized the importance of affective experiences during computer-supported science learning. In particular, studies highlight the interplay between affect and interaction and the significance of positive affect in social interaction on performance. For example, affect and engagement during upper-elementary school collaborative mathematics tasks in small groups showed that positive affect (happy, calm) was related to positive group interactions, while negative affect (tired, tense) was connected to disengagement and social loafing (Linnenbrink-Garcia et al., 2011).

Technology-based inquiry environments can be demanding and require novel ways to deal with the tasks and cope with the requirements of the environments, which highlights the affects directed at the different features of learning environments (Wosnitza and Volet, 2005). Baker et al. (2011) found that the most common affective states for undergraduate pairs in a virtual laboratory for chemistry were engaged concentration and confusion. Graesser et al. (2014) found a larger variety of important learning-centered emotions when students were using advanced learning technologies in computer science, mathematics, physics, and biology topics. Emotions the students found included frustration, boredom, confusion, and engagement/flow as well as moments of happiness, sadness, curiosity, surprise, delight, and anxiety.

Task-related emotions pertain to all learning situations (Wosnitza and Volet, 2005), but are particularly important in learning STEM concepts, which are characterized by varying beliefs about difficulty, anxiety, and lack of control (Pino-Pasternak and Volet, 2018). However, science topics can also arouse positive affect. For example, in a study by Tomas et al. (2016), positive emotions dominated the experiences and perceptions of students when studying socio-scientific issues. In that study, students were able to regulate their negative emotions during the group work to complete the task successfully. King et al. (2017) studied ninth-grade students’ discrete emotions during science activities in chemistry and found that learning new chemistry concepts resulted in frustration but was resolved by revisiting the concepts and through interaction with peers and the teacher.

Inquiry learning processes typically have different phases that can be distinguished from each other. There are different descriptions of the inquiry phases, which—at least partly—reflect the emphasis of deductive or inductive approaches (Pedaste et al., 2015). In their recent review, Pedaste et al. (2015) concluded that a typical inquiry includes phases, such as orientation, conceptualization, hypothesis generation, planning, investigation or experimentation, conclusion, and discussion. These phases have different demands and affordances and require different regulative processes (de Jong and van Joolingen, 1998) that may also arouse different epistemic emotions such as curiosity, joy, confusion, anxiety, frustration, and boredom (Pekrun et al., 2017) but also various social emotions (Wosnitza and Volet, 2005). Although qualitatively different working phases are a fundamental feature of inquiry learning, only a few studies have dealt with affects and motivation during these different phases. Several studies have studied temporal changes in motivation during STEM task performance, but have not specifically analyzed affects and motivation within different phases of inquiry (e.g., Tapola et al., 2013; Rodriguez-Aflecht et al., 2018). An exception is a study by Näykki et al. (2017) in which they organized a collaborative inquiry process into three phases (orientation, intermediate, and reflection) with a script (guiding students’ activities) that included prompts for cognitive and emotional monitoring during the phases (e.g., ‘What kinds of feelings does the task arouse?’). The results showed that student groups expressed more emotional monitoring during the orientation phase than during the two subsequent phases. However, socio-emotional support was provided equally during all phases.

In-depth analyses of discourses in collaborative learning contexts refer to the importance of emotions on the quality of discussion (e.g., exploratory talk, as described by Wegerif and Mercer, 1997) in collaborative groups. High-quality group interaction is possible in groups where participants behave politely and where students feel no shame in expressing ill-structured ideas. In this kind of environment, it is possible to change one’s mind, and there is no aggressive criticism of others’ views. Students do not feel sadness if their initial ideas are not accepted but rather are happy that the collective process led to stronger conclusions that were better justified (Polo et al., 2016). High quality group interaction is, however, not enough if it does not result in successful learning. There is empirical evidence that positive learning and achievement-related emotions predict academic performance (Niculescu et al., 2015). According to recent findings, students’ emotions have an impact on their self-regulated learning and motivation, which for their part effect on students’ academic achievement. However, these findings are focused on individual emotions and learning and are not necessarily directly applicable when collaborative learning in small groups is concerned. Researchers have argued that the role of affects can be dissimilar in situations based on the negotiation of meaning, and require mutual engagement and high levels of social interaction (Linnenbrink-Garcia et al., 2011; Zschocke et al., 2016). Linnenbrink-Garcia et al. (2011) indicated that both neutral and positive affects could facilitate constructive group interactions, while negative affect seemed to hinder productive group interactions.

Study groups consist of individuals and social relations between them. Thus, both individual and social processes require attention, while students jointly regulate motivation and engagement in collaborative learning (Järvelä et al., 2010). Individual group members’ affects can vary because they interpret the cognitive benefits of collaborative work and the organizational-structural group processes and task characteristics differently (Zschocke et al., 2016). The role of individual differences in a group context, as well as the group’s working practices, need consideration to understand better the divergence in group activity and performance (Summers and Volet, 2010). It is possible to analyze the affective tone of interaction on an individual and collective level (e.g., Polo et al., 2016). The aggregated effects of individuals’ affects in groups can be positive or negative, and the way that aggregated affect activates the group work can be high or low (Linnenbrink-Garcia et al., 2011). Many features of the participant’s affective behavior can cause problems for collaborative learning. Individual participating students may be unmotivated or dissatisfied with the tasks (Zschocke et al., 2016), can harm the quality of discussion by aggressive behavior (Polo et al., 2016), or present derogatory remarks about other students (Baker et al., 2013). However, individual participants can also play an important positive affective role in group work: for example, by providing socio-emotional support (Näykki et al., 2017).

According to a common view, affects have different interrelated components, including physiological reactions, subjective experience, and expressive behavior (Gross and Levenson, 1993). This manifold nature of affect highlights the importance of a multimethod approach in studying affects and emotions related to learning. There is a large variety of methods developed and used in studying affects in individual and collaborative learning processes. Methods can be based on snapshots (e.g., questionnaires) before and after learning episodes or measures during learning processes (e.g., observation; Wosnitza and Volet, 2005). Meyer and Turner (2006) have emphasized the importance of comparing and integrating self-reports and observations in the research of classroom practices.

The overall aim of the present study was to contribute to a better understanding of affect in small groups by using a multimethod approach and scrutinizing how group members’ affects and behaviors contributed to the entire group’s collective outcome. Four research questions were generated:

(1) To what extent is affect within a group (valence, intensity) similar at three distinct phases of their collaborative learning activity? In light of limited prior studies of affect within a group at different phases, stages, or different aspects of an activity, the research questions are exploratory in nature.
(2) How is affect within a group (valence, intensity) related to group outcome (high, average, low)? It would be reasonable to expect that group interactions leading to high performance would generate positive affect within the group and the opposite for a group that achieved low performance. However, some studies (e.g., Tomas et al., 2016) have found that fun, collaborative science activities can generate positive emotions that interfere with learning and, in turn, with performance.
(3) What is the degree of within-group consistency in individuals’ affect (valence, intensity) across phases? How does individual affect play out in extreme performing groups and a group displaying within-group diversity and change in individual affect? Previous studies indicate that similar positive affect among individual students would increase collaborative engagement with the task, and vice versa (Linnenbrink-Garcia et al., 2011). There are indications in prior research focused on other types of group processes (such as regulation or roles as indicators of engagement) that individual students have an impact on other group members, thus positively or negatively influencing the group effort, e.g., in terms of initiating and sustaining conceptual talk (Volet et al., 2019; see also, Rogat and Linnenbrink-Garcia, 2011).
(4) How consistent is self-reported affect and observed (researcher-coded) affect at both the group and individual levels? Since self-reports have been considered to serve an overly narrow view of affect (Wosnitza and Volet, 2005), the present study adopted a multimethod approach. In the case of affect, combining self-report data with researchers’ observations of affect during students’ actual collaborative learning processes was expected to provide complementary insight, and therefore a more reliable and richer understanding of affect in a small group regardless of whether the findings concurred or not. Complementarity was expected on the basis that, on the one hand, emotions could be concealed, and thus are not always observable from an external vantage point. While on the other hand, self-assessments can be biased for a range of reasons (e.g., social desirability, weak self-awareness) or affected by a recency effect (assessments were done after each whole session), therefore not providing an adequate account. This fourth question, related to the degree of consistency between self-reported affect and observed affect, was addressed systematically in each of the first three research questions.

## Materials and Methods

### Learning Environment and Research Design

A web-based learning environment, Virtual Baltic Sea Explorer (ViBSE), was designed to offer a realistic context for learning both key science concepts and knowledge integrating biology and chemistry and cultivating scientific practice and reasoning skills (Kinnunen et al., 2018; see also, Vauras et al., 2019). The group activity involved running an experiment on the effects of fast pH changes in very important phytoplankton and certain species of copepods in the Baltic Sea’s food chain, using a dominating science language, i.e., English. ViBSE offered a rich set of tools for students, such as a library of key constructs and phenomena, photos, interviews, and mini-lectures by the crew and researchers of the real research vessel Aranda, laboratory tasks, and links to external web pages concerning the news and state of the Baltic Sea. Thus, during their virtual exploration, the students became acquainted with scientific work, characterized by experimental methods, such as forming hypotheses, simulation of the research design, running experiments, and interpreting and concluding the outcomes. All data underlying the experiments were based on studies by real marine biologists in published articles (see, Bonaglia et al., 2013; Engeström-Öst et al., 2014). During the learning in the virtual learning environment (VLE), students were choosing the topic (in this study they were studying the effects of pH changes on the reproduction of copepods), making hypotheses and study designs and proceeding to the laboratory tasks (choosing the number of sea water bottles, eggs, pH and time). Laboratory tasks consisted of collecting the data, making analyses (e.g., counting the eggs and calculating basic statistics), and concluding and interpreting results. Small group collaborative inquiry, in the role of partners in the marine research team of a real environmental research vessel, was intended to elicit deep-level learning through genuine scientific dialogue and argument. Thus, ViBSE was designed to provide a bridge between the school and science worlds by positioning students as researchers and fostering their adoption of the practices, goals, and methods that guide the authentic research of professional scientists. Since the VLE was new to students and thus challenging for the students in a regular classroom, teacher assistance was further offered when needed (see Koretsky et al., 2019; Vauras et al., 2019).

The learning context for using the VLE were high schools, where students (*N* = 120) worked together in small peer groups (*N* = 39) during their regular science courses at an advanced level, and earned course credits for their participation. The students were between 16 and 19 years (*M* = 17.27; *SD* = 0.68) and over half of them were girls (65%). This gender distribution was related to the course topic, i.e., biology. The teacher assigned students to small peer groups in advance to level the disciplinary knowledge and English language competence within groups. All students were familiar with each other since they studied together in the course. The research team informed and guided teachers in using the VLE and in turn, teachers gave instructions to the students. The students were instructed to collaborate as a team, while the teacher’s role was primarily to scaffold the groups. The small peer groups worked in their own space by their table with a shared laptop, during three sessions lasting 75–95 min each. The three working sessions followed the phases of scientific research: (1) planning: reading materials, generating a hypothesis, and experiment planning; (2) experimentation: including analysis of the results; and (3) conclusions: preparing a group presentation to the class, followed by discussion. All students in the class were asked to complete a paper-and-pencil questionnaire eliciting their affect at the end of each working session to avoid interruptions in the groups’ learning process. This questionnaire was completed individually at all three measurement points, using the same procedure. Participation of both students and teachers was voluntary, and written permission for video recording the groups’ interactions was obtained from all students (and guardians if students were under 18 years of age).

### Participants for the Present Study

Altogether, six intact groups, totaling 18 students (4 boys and 14 girls) were chosen for close analysis out of 39 groups. The decision to select a small number of groups was necessary due to the exploratory nature of the study and, in particular, to allow an in-depth, detailed analysis of each group. These six groups were selected based on their group outcome (two Low, two Average, and two High). Selection criteria for the groups was that they were intact groups of the same three students during the entire process (three working sessions). The outcome measure was the group presentation at the very end of the three working sessions. Two qualified science professionals in biology and chemistry evaluated the overall quality of the groups’ presentations, taking into account the research plan, hypotheses, understanding of the task and presentation structure, actual presentation, conclusions, and quality of the scientific language used in the presentation. The groups were divided into three performance levels based on the quality of their group presentation (1 = low−, 2 = low+, 3 = average−, 4 = average+, 5 = high−, 6 = high+). The number of distinct productive outcome groups was 6 high (13 girls, 3 boys), 14 average (28 girls, 14 boys) and 19 low (34 girls, 22 boys) groups. In this paper, pseudonyms are used to address individual students within the groups.

### Data and Data Analyses

#### Self-Reported Affect

Students were asked to evaluate their affect individually on a systematic affect scale based on the valence of positive and negative affect on a 10-point bipolar Likert scale from the orthogonal positive and negative affective states (e.g., excited-tired, confident-insecure) (Pietarinen et al., 2019). A circumplex model of affect was applied to capture activating and deactivating affects as well as valence (e.g., Feldman Barrett and Russell, 1998; Linnenbrink-Garcia et al., 2011; see also, Scherer, 2005). The selection of affective states for the scale was based on the findings of previous studies and learning-related emotions in advanced learning technologies, as described by Graesser et al. (2014). Each student assessed 12 items altogether, representing 24 affective states (proud-ashamed; enthusiastic-bored; excited-tired; delighted-disappointed; interested-uninterested; confident-insecure; happy-unhappy; glad-angry; pleased-annoyed; satisfied-frustrated; relaxed-anxious; calm-tense). Students’ affect during the group task was measured at the end of each working session, based on their perceptions of the affective states they experienced in each preceding working phase. Aggregated individual reports by a particular group were used in all group-level analyses. For frequency analyses, values ranging from one to four were classified as negative, from five to six as neutral, and from seven to ten as positive. For this study, neutral (total of approximately 22–25% of all self-ratings; see Pietarinen et al., 2019) were excluded from these analyses since the focus was on comparing, specifically, valence and arousal in relation to two different data collection methods (self-reports and observations) and to ensure comparable data for this purpose.

#### Observed Affect

**FIGURE 1:**
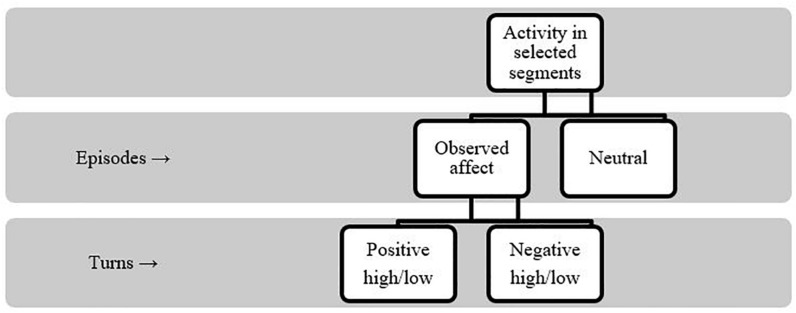
The coding protocol.

The video segments for the in-depth analyses of observed affect were chosen from the total video footage from each group. These segments represented meaningful and continuous verbal interaction (i.e., collaboration) within the group and featured each of the working phases, namely, Planning, Experimentation, and Conclusions, following the steps of scientific research (see Tsovaltzi et al., 2010). Data for coding was thus restricted to these meaningful segments to ensure manageable coding and comparable observation for all groups through all working phases. Because the groups varied in terms of the length of their conversations and activity completion rates, the selected video segments were of unequal length (approximately 10–16 min). Therefore, the group analyses were based on observations appropriate for science learning (see Derry et al., 2010), and two independent coders coded them, using the Observer XT 12. The coding scheme was modified from earlier research on affect dimensions (Scherer, 2005; Linnenbrink-Garcia et al., 2011) and group processes (Vauras et al., 2008; Linnenbrink-Garcia et al., 2011; Rogat and Linnenbrink-Garcia, 2011). Consistent with these prior studies, the researchers’ observations considered both verbal and non-verbal interaction, and paralanguage (sighing, yawning) to capture all possible indicators of affect, i.e., valence and activation. The coding was undertaken first at the episode level (a chain of [verbal] interaction) to define the sequences for turn-level analyses, and then at the turn level (a word, sentence, talk or noticeable gesture of one person) to gain greater insight into the groups’ affect-related interactions. The coding protocol is shown in Figure 1. The total amount of turns across the selected segments was 6390, and 1542 turns (24%) contained the observed affect. The inter-coder agreement was calculated from all turns within randomly selected episodes. Table 1 presents the indicators and examples for diverse levels of valence and intensity.

**TABLE 1:** Affective behavior coding categories and examples.

**Valence**	**Intensity**	**Indicators**	**Examples**
Positive	High	Clear and intense positive gestures, body language or facial expression or specific statement expressing high positive affect, high tone of voice, laughing, and joking while laughing.	Paula: *”Fine, I knew so much”* and laughs, touching her hair. Joel jokes: *”Very reliable research result”* and laughs: *”Somewhere in the university, it is 30 pages.”*
	Low	Clear and light positive gestures, body language, or facial expression or specific statement expressing positive affect, positive tone of voice, smiling, joking with a calm face. Also, an expression of surprise.	Hanna jokes: *”I was maybe avoiding a bit,”* smiling. Anna says: *”Here is a dictionary,”* looking surprised
Negative	High	Clear and intense negative gestures, body language or facial expression, or specific statements expressing high negative affect, high tone of voice.	Isabel says: *”It irritates me when this is in English; everything irritates me now”* (whining) Anna looks irritated: *”What the heck. we have done these slowly”*, turning around
	Low	Clear and light negative gestures, body language, or facial expression or specific statement expressing negative affect, negative tone of voice. Turning away from other(s) with a negative expression in reaction to others or the task. Also, sighing and yawning.	Jesse answers Elias’s comment*: “Some weird organism”* and looks uninterested and amused, watching his mobile phone. Laura says: *”I don’t know”* and yawns.

First, the two coders viewed the selected video segments independently and detected all episodes with observed affect (i.e., verbal interaction between at least two of the three students when affective behavior could be detected). All neutral episodes were excluded from subsequent analyses at the turn level. While the starting and end turns of the episodes identified by the two coders were not always the same, the episodes themselves were located in the same timeframe. The inter-coder agreement initially varied between 64 and 94%, and after discussion, the agreement ranged between 87 and 97%.

Second, both coders coded a random sample of episodes from each group and phase (approximately 30%) independently at the turn level, and agreement for valence and intensity agreement varied between 64 and 92%. All disagreements were minor, concerning mainly differences in intensity (high, low) and occasionally related to valence when sarcasm played a role. After discussion, the agreement varied between 82 and 96%, and the range of Cohen’s kappa-values for all groups and phases (κ = 0.722 – 0.938) were substantial or almost perfect (see Landis and Koch, 1977).

## Results

The results are organized around the first three research questions. The first result addressed the extent of similarities and differences in affect (valence, intensity) in the groups in the three phases of the collaborative learning activity. The second result examined the issue of the relationship between group outcome and affect within groups (valence, intensity), and the third addressed the degree of within-group consistency in individual students’ affect (reported, observed; valence, intensity) across phases. The data used to answer the third research question is complemented by an in-depth narrative analysis of individual affect in three groups: the two extreme performing groups and one group that displayed within-group variation and change in individual affect over the three phases. The fourth question, related to the degree of consistency between self-reported affect and observed affect, is addressed in each of these three questions.

### Similarities and Differences in Affect Within a Group at Three Phases of Their Collaborative Learning Activity (Research Question 1)

**FIGURE 2:**
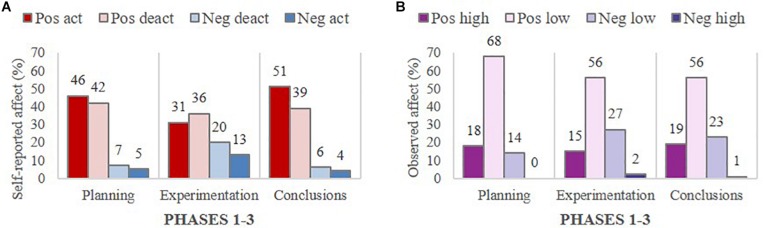
Self-reported **(A)** and observed affect **(B)** overall by valence and intensity (arousal) across three phases.

Self-reported affect within groups (aggregated individual reports) and observed affect within groups (researcher-coded) overall were examined in turn, in terms of valence and intensity. The distribution of self-reported affect by valence and intensity (arousal) across the three phases is presented in Table 2, and the distribution is illustrated in Figure 2A. Further, the distribution of observed affect by valence and intensity (high and low) across the three phases is presented in Table 3, and illustrated in Figure 2B.

**TABLE 2:** Distribution of self-reported affect overall by valence and intensity (arousal) across the three phases.

**Valence**	**Arousal**	**Planning**	**Experimentation**	**Conclusions**
Positive	Activating	77 (46%)	47 (31%)	85 (51%)
	Deactivating	70 (42%)	54 (36%)	66 (39%)
	Deactivating	12 (7%)	31 (20%)	10 (6%)
Negative	Activating	9 (5%)	20 (13%)	7 (4%)
Total		168 (100%)	152 (100%)	168 (100%)

**TABLE 3:** Distribution of observed affect overall by valence and intensity (high and low) across the three phases.

**Valence**	**Intensity**	**Planning**	**Experimentation**	**Conclusions**
Positive	High	113 (18%)	52 (15%)	109 (19%)
	Low	422 (68%)	198 (56%)	320 (56%)
	Low	87 (14%)	93 (27%)	133 (23%)
Negative	High	0 (0%)	8 (2%)	7 (1%)
Total		622 (100%)	351 (100%)	569 (100%)

The findings were interpreted according to percentages because the total number of self-reports of affect was not the same across all phases (168 for Planning and Conclusions, and 152 for Experimentation). As documented in Table 2 and illustrated in Figure 2A, there was a dominance of positive over negative affect overall (high and low arousal combined) in each of the three phases, but the pattern was less salient in the Experimentation phase (67%) than in the Planning phase (88%) and the Conclusions phase (90%). The pattern of self-reported affect in the Experimentation phase differed from the other phases, with one third (33%) being negative, compared to only 12% in the Planning phase and 10% in the Conclusion phase. The relatively high proportion of negative affect during the Experimentation phase may indicate that the task and collaboration with peers were particularly stressful during that phase. It is important to note that students’ self-reported assessment of their affective states at the end of each phase applied to the entire working session and, therefore, while these self-reports reflected their affective state at this particular stage of the task, it may have also reflected their overall mood during that session.

In respect to observed affect and as reported in the method section, 24% of the video data selected for analysis (1542/6390 turns) was identified as containing affect-related behaviors, with the rest considered neutral. This percentage differed only slightly across phases, with 28% (622/2,208 turns) in the Planning phase, 19% (351/1804 turns) in the Experimentation phase, and 24% (569/2,383 turns) in the Conclusions phase.

Overall, the coded observations of students’ affect revealed some similarities and some differences, as found in the self-report data. Table 3 shows the distribution of observed affect overall, by valence and intensity across the three phases; this distribution is illustrated in Figure 2B.

Similar to the self-report data, positive affect was dominant in all phases (over 70%, when combining high and low intensity), but the positive affect in the Conclusions phase was not as salient when based on the observations rather than the self-reports. Furthermore, the positive affect observed in the video data was dominant in low intensity, whereas a high proportion of the self-report data featured positive affect, representing activating rather than deactivating arousal. With regard to the observed negative affect, the percentage was relatively similar to the self-report data for the Planning phase (12% vs. 14%) and the Experimentation phase (33% vs. 29%), but the percentage of observed negative affect was higher than the self-report data for the Conclusions phase (10% vs. 25%).

### Relationship Between Affect Within a Group and Group Outcome (Research Question 2)

**FIGURE 3:**
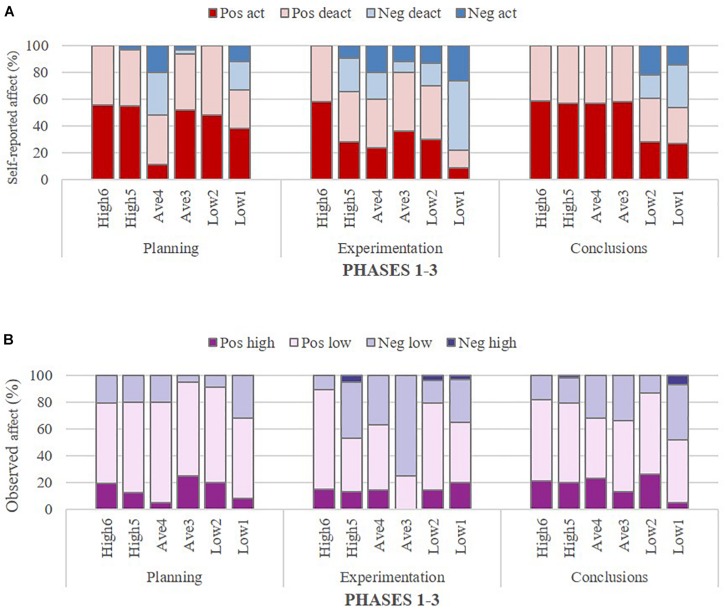
Self-reported **(A)** and observed **(B)** affect across three phases in six distinct outcome groups.

The relationship between affect within a group (valence and intensity) and group outcome was examined by comparing group self-reported (aggregated individual reports) and observed affect (researcher-coded) across the six groups that differed in terms of their group outcome. The distribution of self-reported and observed affect by valence and intensity across the six distinct outcome groups and the three phases, respectively, is presented in Tables 4, 5, and illustrated in Figure 3.

**TABLE 4:** Distribution of self-reported affect in distinct outcome groups across the three phases.

			**Planning**		**Experimentation**		**Conclusions**	
**Group**	**Valence**	**Arousal**	***Number of turns***	***% of turns for group***	***Number of turns***	***% of turns for group***	***Number of turns***	***% of turns for group***
High_6_	Positive	Activating	19	56	14	58	13	59
		Deactivating	15	44	10	42	9	41
		Deactivating	0	0	0	0	0	0
	Negative	Activating	0	0	0	0	0	0
High_5_	Positive	Activating	18	55	9	28	20	57
		Deactivating	14	42	12	38	15	43
		Deactivating	0	0	8	25	0	0
	Negative	Activating	1	3	3	9	0	0
Average_4_	Positive	Activating	2	11	6	24	20	57
		Deactivating	7	37	9	36	15	43
		Deactivating	6	32	5	20	0	0
	Negative	Activating	4	20	5	20	0	0
Average_3_	Positive	Activating	17	52	9	36	21	58
		Deactivating	14	42	11	44	15	42
		Deactivating	1	3	2	8	0	0
	Negative	Activating	1	3	3	12	0	0
Low_2_	Positive	Activating	12	48	7	30	5	28
		Deactivating	13	52	9	40	6	33
		Deactivating	0	0	4	17	3	17
	Negative	Activating	0	0	3	13	4	22
Low_1_	Positive	Activating	9	38	2	9	6	27
		Deactivating	7	29	3	13	6	27
		Deactivating	5	21	12	52	7	32
	Negative	Activating	3	12	6	26	3	14

**TABLE 5:** Distribution of observed affect in distinct outcome groups across the three phases.

			**Planning**		**Experimentation**		**Conclusions**	
**Group**	**Valence**	**Arousal**	***Number of turns***	***% of turns for group***	***Number of turns***	***% of turns for group***	***Number of turns***	***% of turns for group***
High_6_	Positive	High	14	19	15	15	19	21
		Low	45	60	74	74	56	61
		Low	16	21	11	11	16	18
	Negative	High	0	0	0	0	0	0
High_5_	Positive	High	6	12	7	13	18	20
		Low	34	68	22	40	51	59
		Low	10	20	23	42	17	19
	Negative	High	0	0	3	5	2	2
Average_4_	Positive	High	3	5	7	14	10	23
		Low	41	75	24	49	20	45
		Low	11	20	18	37	14	32
	Negative	High	0	0	0	0	0	0
Average_3_	Positive	High	49	25	0	0	15	13
		Low	135	70	3	25	61	53
		Low	10	5	9	75	39	34
	Negative	High	0	0	0	0	1	1
Low_2_	Positive	High	35	20	10	14	44	26
		Low	121	71	45	65	104	61
		Low	15	9	11	17	22	13
	Negative	High	0	0	3	4	0	0
Low_1_	Positive	High	6	8	13	20	3	5
		Low	46	60	30	45	28	47
		Low	25	32	21	32	25	41
	Negative	High	0	0	2	3	4	7

Overall, as displayed in Table 4 and illustrated in Figure 3, all six outcome groups reported dominantly positive arousal (activating/deactivating combined) for all phases, with two exceptions: Average_4_ for the Planning phase (only 48%), and Low_1_ for the Experimentation phase (only 22%). The two extreme groups, High_6_ and Low_1_, were strikingly different in their self-reported affect; as the highest performing group, High_6_ systematically reported positive arousal (activating/deactivating combined) for each phase (100%), whereas the lowest performing group, Low_1_, reported a substantial proportion of negative arousal (activating/deactivating combined) for each phase (Planning 33%, Experimentation 78%, and Conclusions 46%). This contrasting pattern of findings is consistent with the performance-related differences between these groups. The pattern is also consistent with anecdotal statements made by members of group Low_1_, who repeatedly expressed uncertainty regarding the task and their performance. Furthermore, it was noteworthy that all high and average performing groups reported strictly positive arousal (100%) for the Conclusions phase, whereas the two lowest performing groups, Low_1_ and Low_2_, reported considerably high negative arousal (activating/deactivating combined) for the Conclusions phase (Low_2_ 39%; Low_1_ 46%). Apart from the two extreme groups, the group Average_4_ attracted the researchers’ attention and further examination [see Section Degree of Within-Group Consistency in Individual Affect Across Phases: Insights From Three Illustrative Groups? (Research Question 3)], since this group reported a substantial proportion of negative arousal (activating/deactivating combined) for the Planning phase (42%) and the Experimentation phase (40%), but no negative arousal for the Conclusions phase (0%).

In respect to the findings on observed affect, as shown in Table 5 and illustrated in Figure 3, positive intensity (high and low combined) was also dominant across groups and for all phases but for one exception, Average_3_ for Experimentation (only 25%). In contrast to the self-reports, this group displayed 75% negative affect (although of low intensity) during the Experimentation phase, while their self-reports showed only 20% negative affect. Overall, and despite the dominantly positive observed affect, some negative affect was also observed in all groups, irrespective of the group outcome. However, it is important to note that only a few groups displayed high intensity negative affect; most groups’ observed negative affect was of low intensity.

Interestingly, the two extreme groups’ (High_6_ and Low_1_) self-reported and observed affect revealed similar within-group differences. Furthermore, the two extreme groups’ differences in observed affect were not as striking as the differences in their self-reported affect. Specifically, while High 6 reported exclusively positive affect, the coded observations of their interactions displayed some negative affect, though of low intensity and ranging only from 11 to 21%. In contrast, Low_1_ displayed predominantly positive affect (from 55 to 68%) and very little negative affect of high intensity (only 0 to 7%). Altogether, regarding the lowest performing group (Low_1_), the proportion of observed negative affect was remarkably similar to their self-reported affects for both the Planning phase (self-reported affect 33%; observed affect 32%) and the Conclusions phase (self-reported affect 46%; observed affect 48%). The intensity of negative affect, however, appeared lower in the observed affect data than the self-reported data. For the Experimentation phase, the proportion of negative affect was higher and of higher intensity in the self-report than in the observed data (self-reported affect 78%; observed affect 35%).

Alongside the extreme groups and considering their observed affect, Average_4_ continued to represent a group of interest, as their observed negative affect was not as noticeably high (20 and 37%, respectively) as their self-reported negative affect for Planning and Experimentation (52 and 40%, respectively). Although there were observations of negative affect in the Conclusions phase, only positive affect was self-reported at that phase. Exploring further at the individual level why this group may have reported predominantly positive affect in the Conclusions phase when a third of their turns (32%) in the segment selected for analysis displayed some negative affect seemed warranted and is reported in Section “Degree of Within-Group Consistency in Individual Affect Across Phases: Insights From Three Illustrative Groups? (Research Question 3).”

All in all, the general finding regarding self-reported and observed affect within groups indicates that positive and negative affect appeared to be of lower intensity in the observations than in the self-reports. However, the observations revealed negative (low intensity) affect in many instances where it was not reported in self-assessments. These general findings, obtained by applying two distinct methods, will be discussed later. Before that, the investigation of affect within a group will be further deepened through illustrations of self-reported and observed affect at the individual level within three distinct outcome groups (High_6_, Low_1_, and Average_4_).

### Degree of Within-Group Consistency in Individual Affect Across Phases: Insights From Three Illustrative Groups? (Research Question 3)

**FIGURE 4:**
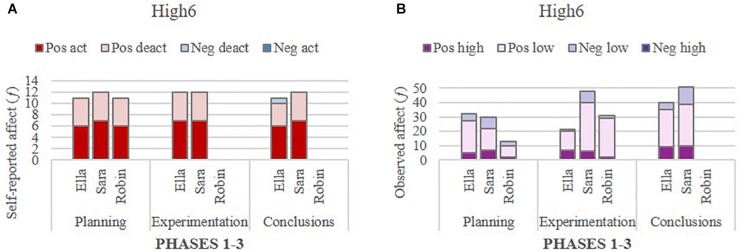
Self-reported affect **(A)** and observed **(B)** affect in the group High_6_.

**FIGURE 5:**
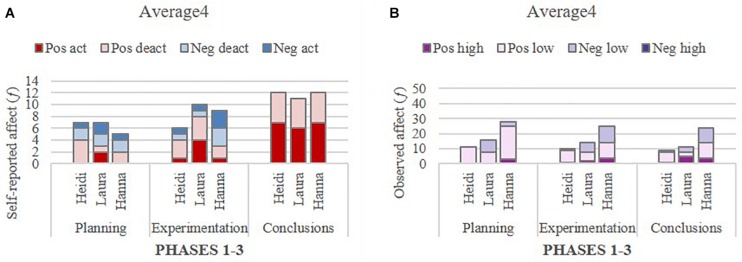
Self-reported **(A)** and observed **(B)** affect in the group Average_4_.

**FIGURE 6:**
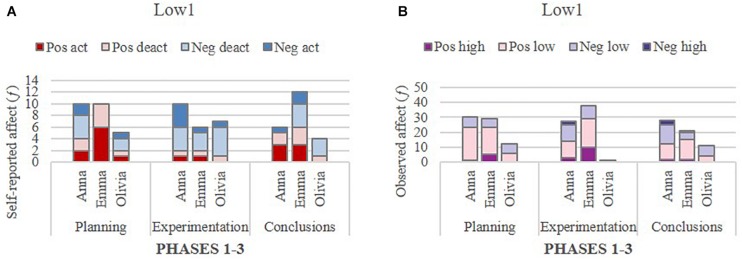
Self-reported **(A)** and observed **(B)** affect in the group Low_1_.

To gain insight into the within-group dynamics of affect, a glance at the degree of homogeneity in individual self-reported and observed affect within groups revealed rather consistent affect patterns among members in the two extreme groups, but not in the Average_4_ group. The self-reported affect and the observed affect of individuals within their respective groups are presented in Figures 4–6, followed by excerpts from their verbal interactions related to the task and science content.

In the highest performing group (High_6_), the reported and observed affect of all individual students were predominantly positive, and in the lowest performing group (Low_1_), all students reported and were observed to display a substantial degree of negative affect. In the group of interest, Average_4_, the patterns appeared more complex and varied. Each group is presented in turn below.

It is important to note that when coding observed affect, high and low positive intensity was determined based on a composite of smiling, laughing, and joking about the activity, content, or technical issues, and sometimes the attitudes, interest, and level of content knowledge could be detected from students’ comments. In respect to the high and low levels of negative intensity, there was a greater variety of indicators, for example, irritation, bitterness, tiredness, frustration, and boredom, which occurred alongside negative gestures and facial expressions (such as sighing or yawning). One challenge was to code sarcastic comments, as the intended meaning was often found to be ambiguous.

Group High_6_ (Ella, Robin, Sara): Students in this group reported mainly positive affect and hardly any negative affect. Only Ella reported tiredness (low negative arousal) at the Conclusions phase. However, this exception is unlikely to have had any impact on group outcome. In contrast, some negative affect was observed in each video segment selected for coding. It is noteworthy that high intensity negative affect was totally absent in both self-reports and observations, as shown in Figure 4. Positive affect (high and low intensity combined) was dominant in the observations of all students; for Ella, it ranged between 84 and 95% (*M* = 89%), for Sara 73–83% (*M* = 77%), and Robin 77–94% (*M* = 86%), indicating that Ella displayed the most positive affect across the three phases (see Figure 4). Remarkably, humor and laughter were present seamlessly in all the conversations related to the scientific content of the task, as illustrated in the verbal interaction example of positive (high and low intensity combined) affect at the Conclusions phase. The group was writing the interpretation of the results and searching for information about the effects of pH on copepods and changes in the food chain from the internet:

Ella: “It depends on the species”…“We cannot know what species there should be”
Sara [whispers]: “Nauplius … here!” smiling, surprised, and delighted: “The larva of the crustacean,” smiling and looking at Ella, then laughing with her
Ella: [with a higher voice] “Yes, wonderful!” looking satisfied
Ella: “And now when it translated it in Finnish it was just right,” laughing

Students in group High_6_ demonstrated high levels of concentration and a determination to complete the task. They focused mainly on the task, and when off-task behavior occurred, it ended quickly, and the group returned to the task. Robin was absent twice during the working periods, and assessed his affect only once, but he was present twice in the selected segments for observations because the group proceeded quickly to the Experimentation phase in the first working period. Robin’s attention was often focused on the technical details connected to experimentation in the virtual laboratory, but despite some skeptical questioning, he managed to keep his tone humorous and thus positive, as the following example at experimentation phase of positive low intensity affect demonstrates. The teacher was helping the group to build the experimental design of the study, the number of water bottles, selecting the pH, selecting the time for egg development, and calculating the number of eggs:

Robin: “The number of bottles … what’s the point of that?” smiling and amused
Ella and Sara are looking at Robin and smiling
Sara: “Shall we put here the number of the bottles too?”
Ella and Robin are still smiling
Robin: “Why does it matter how many bottles there are?” smiling
Sara: “I don’t know,” shaking her head and laughing

In respect to Robin’s lack of self-reported data, and based on the finding of relatively equally distributed positive affect within this group, it is reasonable to assume that his absence had minimal impact on the group outcome and the overall affect within the group. From the very beginning of the collaboration, individual participation appeared equal, and the group atmosphere was very open and positive, thus inviting anyone to join the conversation. Interactions were mainly polite and respectful, without any rude or disrespectful comments to other group members. The few negative comments were mainly directed at the task, the content or technical issues concerning the learning environment, which means that the positive tone of the verbal interaction was maintained, as seen in the following example of low negative intensity affect at the experimentation phase. The students had problems to understand what to do with the experimental design, and then the teacher arrived:

Sara: “Umm, difficult to find this kind of pH value…”
Robin: “It is something like six and a half,” frowning
Then, everyone is smiling

Group Average_4_ (Hanna, Heidi, Laura): In contrast to group High_6_, the group Average_4_ appeared to lack interest and motivation for the task and achieving good performance, even though they displayed continued concentration throughout the activity. In support of this claim was the substantially high proportion of negative affect reported by each student in the Planning and Experimentation phases: Heidi, 43 and 33%; Laura, 57 and 20%; Hanna, 60 and 67% (see Figure 5). Markedly, the important negativity emerging from these self-reports disappeared in the Conclusions phase, since all students reported dominantly positive affect and no negative affect. Comparing students’ self-reports of affect with the researchers’ observed affect revealed a more complex and diverse picture of affect within this group. Contrary to students’ self-reports, the observed affect showed evidence of the dominance of positive affect for all students across the three phases. Specifically, as illustrated in Figure 5, for Heidi, positive affect (low intensity) varied between 89 and 100% (*M* = 93%), for Laura between 50 and 72% (*M* = 60%) and for Hanna between 56 and 89% (*M* = 68%). There were only a few exceptions where negative affect was dominant for Laura, such as self-report at the Planning phase and observations in the Experimentation phase, and for Hanna, self-report at the Planning and Experimentation phases. One possible explanation may be that the task was perceived as not interesting or challenging enough. The following excerpt illustrates the visibility of the negative tone in the comments related to the positive (high and low intensity combined) affect in the group’s verbal interaction at the conclusions phase, when the students were discussing and writing the interpretation of the results, and making the presentation:

Laura: “Yes, yes … what should I put here now?” smiling and laughing
Hanna: “A wild guess,” laughing with Heidi
Laura: “It stays there, closely,” laughing, “Well… hmm,” smiling
Hanna: “I don’t know, figure out something better,” smiling
Laura: “I think that was good,” laughing
All three are laughing

The negative tone was visible in the comments related to the positive (high and low intensity combined) affect in the group’s verbal interaction at the experimentation phase as well, while the students were discussing how to start the experiment and proceed:

Heidi: “Do we study everything now?” smiling
Hanna: “Everything,” laughing
Heidi [continues]: “Like eggs, hatching … Do we study everything?” smiling
Laura: “Umm,” smiling

In respect to negative affect, and contrast to students’ self-reports for the Planning and Experimentation phases, there was no observable high intensity negative affect. The self-reports highlighted mainly tiredness and frustration, but also insecurity (Heidi and Laura) as well as anger and annoyance (Hanna). Despite some negative tone of affect in the observations, participation in the group appeared equal, the students were friendly and kind to each other, and humor and joking was mainly directed at the task or the technology but not at other students in the group. The following excerpt at the Conclusions phase is a conversation where the students were commenting on the task (low negative intensity affect), illustrating how low motivation and attitude may have contributed to the average performance of this group when the students were writing the interpretation of the results and making the presentation:

Laura is sighing [loudly]
Hanna: “I don’t get why we have to write these here,” looking tired and bored, leaning into her hand, sighing
Heidi: “Yeah”
Hanna is sighing [loudly]

Group Low_1_ (Anna, Emma, Olivia). In contrast to groups High_6_ and Average_4_, where self-reported and observed affect were not entirely consistent, group Low_1_ reported a dominance of negative affect (high and low intensity combined) across all three phases, and the same finding was obtained in the analyses of their interactions. Positive affect was therefore limited within this group; specifically, Anna reported 20–83% (*M* = 48%) positive affect (high and low intensity combined), Emma 33–100% (*M* = 61%) (high and low intensity combined), and Olivia 14–40% (*M* = 26%) (low intensity). In respect to the observations, a more positive picture of affect emerged compared to self-report. For example, the observations of Anna revealed 43–77% (*M* = 57%) positive affect (high and low intensity combined), of Emma 71–80% (*M* = 76%) positive affect (high and low intensity combined), and of Olivia 36–100% (*M* = 62%) positive affect (low intensity). Based on the video observations, negative communication within the group was visible, as illustrated in Figure 6. As can be seen, Olivia displayed the lowest proportion of positive affect in this group, but she also had the smallest number of turns, since during the selected episodes representing meaningful and continuous verbal interaction (see Section Materials and Methods), she did not participate much in the verbal interactions and often sat quietly, looking at the screen.

In comparison to groups High_6_ and Average_4_, where all group members participated relatively equally, in group Low_1_ it was Anna, the weakest student (based on a rather weak grade in the science course) who led the group; thus, members’ participation was not equal. It was evident that Anna and Emma were ignoring Olivia, possibly because she was, voluntarily or not, quiet most of the time in the segments selected for observation. Moreover, although students in this group concentrated on the task, they did not seem to understand what had to be done. Overall, students in this group appeared to be passive, confused, and worried. Confusion and helplessness were visible even when the conversation displayed positive (high and low intensity combined) affect, as shown in the following excerpt at the planning phase when the group was searching information about the Nauplii from the internet:

Anna: “What is this?” looking at the screen
Emma: “I don‘t know, click there so we can get away from here,” laughing
Anna and Olivia are smiling
Anna: “Let’s do so,” smiling
Emma: “So you did click then,” laughing [widely] and looking at Anna
Anna: “Yeah,” smiling

Initially, the group appeared to work on the task from a positive position but as the phases evolved, affect changed from positive to a more negative tone. At the end of the first phase, their self-reports displayed interest and calmness, but only calmness was sustained in the next two phases. The observed affect was more directed at themselves than at the task or the technology like the other groups, and they did not appear to know where they should be heading. Details and irrelevant matters captured their attention, and they often lost a sense of direction. Finally, nobody in the group appeared interested in completing the task, as illustrated in the positive low intensity affect example at the conclusions phase, when the group was making their presentation and accidentally clicking a new tab:

Anna: “Here is a dictionary,” looking surprised
Emma: “What the damn is this? You don‘t say…,” smiling

Furthermore, the negative affect increased over time within this group as students appeared to realize the inevitable failure of their assignment. They reported a wide array of negative affective states, such as tiredness, boredom, frustration, disinterest, disappointment, and insecurity. One noticeable observation in this group compared to the other groups is that two students, Emma and Olivia, reported feeling ashamed in the Conclusions phase, as they realized that they failed the task. As the weakest student, Anna did not report being ashamed, but one may speculate that she was perhaps used to failing and thus accepted the poor group outcome at the end. Eventually, this group asked for help from the teacher because they were not able to proceed with the task any more. However, they did not understand the teacher’s instruction and failed to complete the task, as reflected in the negative affect (high and low intensity combined) example presented below at the experimentation phase, while examining the questions concerning the design of the study:

Anna: “What the heck … we have done these slowly,” turning around and looking at the other groups
Emma: “You don’t say,” smiling and waving her hands

The negative affect (high and low intensity combined) was present in the group discussion concerning the main variables in the study as well as the rules of scientific reasoning at the experimentation phase:

Anna: “I don’t understand this at all,” shaking her head, looking worried
Emma is smiling and mumbling something [uncodable]
Anna [leaning forward on the table]: “I don’t understand,” looking desperate
Emma: “I don’t understand either,” laughing

## Discussion

### Similarities and Differences in Affect Within Groups at Three Phases of Their Collaborative Learning Activity

Examining affect in collaborating groups in three working sessions showed how positive affect was prevalent across all three learning phases, as evidenced by both methods, self-reports, and observations (RQ1). Positive affect was dominant even though the students worked with a challenging science task in an unfamiliar, web-based VLE. This pattern of finding was obtained using two distinct methods of data collection and analysis, self-reports, and video observations. This finding is in line with other studies that reported the dominance of positive emotions in students’ experiences and perceptions of science learning, corroborated by self-reports, video observations, and interviews (Linnenbrink and Pintrich, 2004; Tomas et al., 2016).

In addition, the patterns of self-reported affect in the Experimentation phase were found to differ from the other phases by showing a relatively high proportion of negative affect. This pattern may indicate a more stressful and demanding phase in students’ process of science learning. In this particular activity, handling the experimentation was quite different from the simple, hands-on laboratory tasks students had performed earlier in their studies. An opposite pattern was noticed in the last phase, Conclusions, where self-reports displayed overriding positive affect but observed affect not to this extent. This outcome is consistent with the study by King et al. (2017), as they found that in respect of the challenges when students were able to work with the science content, they displayed positive emotions. It is also plausible to assume that in this instance, the selection of the analyzed video segments may have played a role. The observations were made when students were in the process of completing the initial work on the content of the presentation (outcome), whereas self-reports elicited students’ affect experienced throughout the whole session (preparing, writing, and presenting). Thus, it is possible that self-reports of positive affect at the Conclusions stage captured students’ relief that they had completed this challenging collaborative group assignment.

### Relationship Between Affect in the Groups and the Group Outcome

The relationship between affect within groups and group outcome proved to be more complicated than indicated by earlier literature emphasizing the impact of positive affect on science activities (e.g., Laukenmann et al., 2003) and achievement (Ahmed et al., 2013; Liu et al., 2014). This expected effect of positive vs. negative affect on performance was only evident in extreme groups, with the highest performing group showing consistent and dominant positive affect and the lowest performing group, to a noticeable degree, negative affect. The finding concerning these extreme groups is consistent with Linnenbrink-Garcia et al.’s (2011) study, which found that positive group interactions were associated with positive affect and negative affect resulted in disengagement and social loafing. Overall, however, the outcomes resonated with the multimethod study in mathematics in which the students of distinct outcome groups (high, moderate, low) experienced both positive and negative emotions, regardless of the difficulty level of tasks (Ahmed et al., 2013).

Furthermore, findings from the groups other than the extreme groups of the present study revealed that the proportion of affect was not systematically related to the groups’ performance. Positive affect probably did not always stem from engagement with the task and scientific content, but also from students’ social interactions with their peers or superficial features of the technology (see also, Wosnitza and Volet, 2005). This finding leads to a tentative conclusion that experiencing positive or negative affect may not always be directly related to performance or learning quality (process or product); this outcome has been reported in previous studies (Tomas et al., 2016; see also, Fredrickson, 1998). For example, Tomas et al. (2016) found that fun-related emotions are not conducive to productive learning because they interfered. Although Pietarinen et al. (2019) showed that joviality, indicating, e.g., joy, interest, and enthusiasm, positively related to the level of group outcome and mediated by aiming for scientific understanding, their findings highlighted strongly the role of more clearly task-oriented affect, namely self-assurance, which is composed of confidence and pride. In future research, the source of fun-related or joy-related emotions and their relationship to the quality of learning should be investigated.

### Degree of Within-Group Consistency in Individual Affect (Valence, Intensity) Across Phases

Although the degree of participation of individual group members varied, their affect echoed rather well with each other. Three groups of interest (two extreme groups and one average group with an evolving affect pattern) were brought under great scrutiny to deepen our understanding of how individual group members play a role in collaborative work. In the highest performing group, all students reported only positive and displayed positive and very little (low intensity) affect. Sustained positive tone in this group signposted the importance of shared affect and therefore can be interpreted in terms of the mutual provision of socio-emotional support (Näykki et al., 2017; see also, Pietarinen et al., 2019). In the lowest performing group, although all students experienced and displayed both positive and negative affect, the degree of negative affect was notable. The discourse illustrations from this group indicate that the students struggled with the task demands; one strong indication of their struggle was their expressed feelings of shame and their inability to benefit from teacher support. Their observed affect, though, indicated a more positive tone, and it is possible that it was their social interactions, not the challenging and possibly frustrating task, that triggered their positive emotional behavior, whereas their reported mood after the whole session was more negatively clouded. This interpretation would be consistent with earlier outcomes showing that if students are appropriately challenged according to their skills, they will more likely feel positive (Schneider et al., 2016), whereas unresolved obstacles may ultimately lead to boredom and disengagement (D’Mello and Graesser, 2012).

Interestingly, all individual students in the average performing group that were examined closely showed some movement, from stating varying degrees of negative affect in the first two sessions to reporting overriding positive affect in the last one. Although a hint of negativity was evident in the researchers’ observations of their last session, their self-reported affect at the end was very positive for all of them. Illustrations from their discourse indicate frustration and boredom with the task, but not with their social interactions as such. One can only speculate that their positive experiences at the end captured a feeling of relief after ending the task or their satisfaction that they were eventually able to complete the task. The patterns of affect displayed within this group throughout a challenging collaborative activity raise the complex issue of the relationship between affect, engagement, and learning. Since these types of more or less average performing groups are the most typical in educational settings, future research should go beyond comparisons of extreme performing groups and try to unveil the affective processes and their effect on learning of the most typical, often very diverse, groups.

## Conclusion

This study analyzed the affective tone of interactions at both group and individual level (see, Polo et al., 2016) by comparing and integrating self-reports and observation as emphasized by Meyer and Turner (2006). Further, following Linnenbrink-Garcia et al. (2011), positive and negative affect, i.e., valence, as well as the intensity of affect in terms of high and low were assessed and analyzed from both individuals’ separate and group-level aggregated affects.

Overall, the two methods resulted in highly corroborated outcomes, but the intensity of affect appeared stronger in self-reports than in observations. This result seems to concur with the premise of invisible emotions stated in the research questions. As the students in this study were not young children, the social expectations for adult-like, task-oriented behaviors are presumably influencing their interaction in formal learning contexts. In particular, the influence of age is supported in the observed data showing that neutral, non-affective interaction and behavior was prevailing. It further needs to be kept in mind that the observations focused on visible affective behavior *in situ* in the restricted context of meaningful task-related episodes, whereas self-reports covered the whole session retrospectively and could be based on more salient or recent feelings and memories. From this viewpoint, the matching outcomes concerning the valence of affect strengthen the reliability and consistency of outcomes. Thus, self-reports and observations in this study show that they can be used as measures of students’ affect in learning situations, either separately or in combination. Both measures showed similar results underlining the dominance of positive affect in all phases regardless of the measurement point or instrument. Also, in combination, these measures can supplement the scope of the analyses by presenting a more comprehensive understanding of the phenomenon under study.

Finally, caution with generalizations is warranted, since the focus of this study was in high school students and particularly in six groups of science education students, which limits the generalizability of the results. However, despite this limitation, the outcomes of this study at the group and individual level also raise challenges for future studies by pointing out the complex dynamics of affect, task engagement, and achievement in average groups. Prior research has strongly focused on extreme groups, which also in this study showed the least complicated associations between these factors.

Since learning, whether in traditional or novel (e.g., virtual) environments, is rarely solely student-led at school contexts, the role of the teacher, as a resource for scientific inquiry in providing not only cognitive but importantly also affective support, should be investigated (see also, Vauras et al., 2019). Although this was outside the scope of the present study, the discourse examples revealed that stronger teacher support would have been needed, particularly in facing task challenges and in the case of struggling groups where students probably lack confidence in science (see also, Volet et al., 2019).

## Data Availability Statement

The datasets for this manuscript are not publicly available because the participants are identifiable in the video data. Requests to access the datasets should be directed to MV, Vauras@utu.fi.

## Ethics Statement

The studies involving human participants were reviewed and approved by The Ethics Committee of the University of Turku. Written informed consent to participate in this study was provided by the participants’ legal guardian/next of kin. Written informed consent was obtained from the individual(s), and minor(s)’ legal guardian/next of kin, for the publication of any potentially identifiable images or data included in this article.

## Author Contributions

TP was responsible for data acquisition and analysis, and drafted the preliminary version of the manuscript. MV, SV, and EL contributed to the writing. All the authors were involved in editing of the manuscript and approved the final manuscript.

## Conflict of Interest

The authors declare that the research was conducted in the absence of any commercial or financial relationships that could be construed as a potential conflict of interest.
